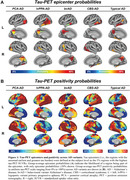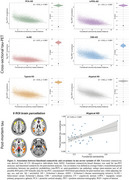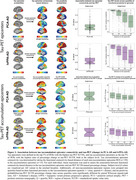# Heterogeneous tau patterns in atypical AD are explained by connectivity‐associated tau progression

**DOI:** 10.1002/alz70856_104871

**Published:** 2026-01-07

**Authors:** Hannah de Bruin, Colin Groot, Alzheimer's Disease Neuroimaging Initiative (ADNI), Henryk Barthel, Gérard N Bischof, Ganna Blazhenets, Ronald Boellaard, Baayla D.C. Boon, Matthias Brendel, David M Cash, William Coath, Gregory S Day, Brad C. Dickerson, Elena Doering, Alexander Drzezga, Christopher H van Dyck, Thilo van Eimeren, Wiesje M. van der Flier, Carolyn A Fredericks, Tim D Fryer, Elsmarieke van de Giessen, Brian A. Gordon, Jonathan Graff Radford, Lea T. Grinberg, Oskar Hansson, Diana A Hobbs, Günter U Höglinger, Merle C Hönig, David J. Irwin, P Simon Jones, Keith A. Josephs, Yuta Katsumi, Renaud La Joie, Eddie B. Lee, Johannes Levin, Maura Malpetti, Scott M McGinnis, Adam P Mecca, Rosaleena Mohanty, Ilya M. Nasrallah, John T O'Brien, Ryan S O'Dell, Carla Palleis, Robert Perneczky, Jeffrey S Phillips, Deepti Putcha, Gil D. Rabinovici, Nesrine Rahmouni, Pedro Rosa‐Neto, James B Rowe, Michael Rullmann, Osama Sabri, Dorothee Saur, Andreas Schildan, Jonathan M Schott, Matthias L Schroeter, William W. Seeley, Stijn Servaes, Irene Sintini, Ruben Smith, Salvatore Spina, Jenna Stevenson, Erik Stomrud, Olof Strandberg, Joseph Therriault, Pontus Tideman, Alexandra Touroutoglou, Anne E Trainer, Denise Visser, Fattin Wekselman, Philip SJ Weston, Jennifer L. Whitwell, David A. Wolk, Keir X X Yong, Yolande A.L. Pijnenburg, Nicolai Franzmeier, Rik Ossenkoppele

**Affiliations:** ^1^ Alzheimer Center Amsterdam, Neurology, Vrije Universiteit Amsterdam, Amsterdam UMC location VUmc, Amsterdam, Netherlands; ^2^ Institute for Stroke and Dementia Research, Klinikum der Ludwig‐Maximilians Universität München, Munich, Germany; ^3^ Amsterdam Neuroscience, Neurodegeneration, Amsterdam, Netherlands; ^4^ Laboratory of Neuro Imaging (LONI), University of Southern California, Los Angeles, CA, USA; ^5^ Leipzig University Medical Center, Leipzig, Germany; ^6^ University of Cologne, Faculty of Medicine and University Hospital Cologne, Department of Nuclear Medicine, Cologne, Germany; ^7^ Memory and Aging Center, Weill Institute for Neurosciences, University of California San Francisco, San Francisco, CA, USA; ^8^ Amsterdam University Medical Center, Amsterdam, Netherlands; ^9^ Mayo Clinic, Jacksonville, FL, USA; ^10^ Institute for Stroke and Dementia Research (ISD), University Hospital, LMU, Munich, Bavaria, Germany; ^11^ UK Dementia Research Institute at UCL, London, United Kingdom; ^12^ Dementia Research Centre, UCL Queen Square Institute of Neurology, University College London, London, United Kingdom; ^13^ Mayo Clinic in Florida, Jacksonville, FL, USA; ^14^ Frontotemporal Disorders Unit and Massachusetts Alzheimer's Disease Research Center, Department of Neurology, Massachusetts General Hospital and Harvard Medical School, Boston, MA, USA; ^15^ Research Center Jülich, Institute for Neuroscience and Medicine II, Molecular Organization of the Brain, Jülich, Germany; ^16^ Alzheimer's Disease Research Unit, Yale School of Medicine, New Haven, CT, USA; ^17^ University of Cologne, Faculty of Medicine and University Hospital Cologne, Department of Neurology, Cologne, Germany; ^18^ Yale School of Medicine, New Haven, CT, USA; ^19^ University of Cambridge, Cambridge, United Kingdom; ^20^ Department of Radiology and Nuclear Medicine, Amsterdam UMC, Vrije Universiteit Amsterdam, Amsterdam Neuroscience, Amsterdam, Netherlands; ^21^ Department of Radiology, Washington University School of Medicine, Saint Louis, MO, USA; ^22^ Mayo Clinic, Rochester, MN, USA; ^23^ Memory and Aging Center, UCSF Weill Institute for Neurosciences, University of California, San Francisco, San Francisco, CA, USA; ^24^ Clinical Memory Research Unit, Lund University, Malmö, Skåne, Sweden; ^25^ Department of Radiology, Washington University in St. Louis, St. Louis, MO, USA; ^26^ Munich Cluster for Systems Neurology (SyNergy), Munich, Bavaria, Germany; ^27^ University of Pennsylvania, Philadelphia, PA, USA; ^28^ Department of Neurology, Mayo Clinic, Rochester, MN, USA; ^29^ Department of Pathology & Laboratory Medicine, Perelman School of Medicine, University of Pennsylvania, Philadelphia, PA, USA; ^30^ Department of Neurology, LMU University Hospital, LMU Munich, Munich, Munich, Germany; ^31^ Frontotemporal Disorders Unit and Massachusetts Alzheimer's Disease Research Center, Department of Neurology, Massachusetts General Hospital and Harvard Medical School, Boston, MA, USA; ^32^ Alzheimer's Disease Research Unit, Yale University School of Medicine, Department of Psychiatry, New Haven, CT, USA; ^33^ Division of Clinical Geriatrics, Centre for Alzheimer Research, Karolinska Institutet, Stockholm, Sweden; ^34^ Department of Psychiatry, University of Cambridge, Cambridge, United Kingdom; ^35^ University Hospital, LMU Munich, Munich, Germany; ^36^ Department of Psychiatry and Psychotherapy, University Hospital, LMU Munich, Munich, Germany; ^37^ Penn Frontotemporal Degeneration Center, Department of Neurology, Perelman School of Medicine, University of Pennsylvania, Philadelphia, PA, USA; ^38^ Frontotemporal Disorders Unit, Department of Neurology, Massachusetts General Hospital and Harvard Medical School, Boston, MA, USA; ^39^ McGill University, Montreal, QC, Canada; ^40^ Department of Clinical Neurosciences and Cambridge University Hospitals NHS Trust and Medical Research Council Cognition and Brain Sciences Unit, University of Cambridge, Cambridge, United Kingdom; ^41^ Department of Nuclear Medicine, University of Leipzig, Leipzig, Germany; ^42^ Department of Neurology, University of Leipzig, Leipzig, Germany; ^43^ University College London, London, England, United Kingdom; ^44^ Clinic for Cognitive Neurology, University of Leipzig, Leipzig, Germany; ^45^ Department of Pathology, University of California, San Francisco, San Francisco, CA, USA; ^46^ McGill Centre for Studies in Aging, Department of Neurology and Neurosurgery, McGill University, Montreal, QC, Canada; ^47^ Department of Radiology, Mayo Clinic, Rochester, MN, USA; ^48^ Clinical Memory Research Unit, Department of Clinical Sciences Malmö, Faculty of Medicine, Lund University, Lund, Sweden; ^49^ Clinical Neurosciences Imaging Center, Yale University School of Medicine, New Haven, CT, USA; ^50^ Radiology & Nuclear Medicine, Vrije Universiteit Amsterdam, Amsterdam UMC location VUmc, Amsterdam, Netherlands; ^51^ Memory and Aging Center, UCSF Weill Institute for Neurosciences, University of California San Francisco, San Francisco, CA, USA; ^52^ Dementia Research Centre, UCL Queen Square Institute of Neurology, London, United Kingdom; ^53^ Department of Neurology, Perelman School of Medicine, University of Pennsylvania, Philadelphia, PA, USA; ^54^ Alzheimer Center Amsterdam, Department of Neurology, Amsterdam UMC, location VUmc, Amsterdam, Netherlands

## Abstract

**Background:**

The link between regional tau load and clinical manifestation of Alzheimer's disease (AD) highlights the importance of characterizing spatial tau distribution. In typical (memory‐predominant) AD, the spatial progression of tau pathology mirrors the functional connections from temporal lobe epicenters. However, atypical (non‐amnestic‐predominant) AD variants with heterogeneous tau patterns provide a key opportunity to assess the universality of connectivity as a scaffold for tau progression.

**Method:**

We included tau‐PET data from 320 subjects with atypical AD, characterized by highly heterogeneous tau patterns (*n* = 139 posterior cortical atrophy/PCA‐AD; *n* = 103 logopenic variant primary progressive aphasia/lvPPA‐AD; *n* = 35 behavioural variant AD/bvAD; *n* = 43 corticobasal syndrome/CBS‐AD) from 14 sites, with a subset of patients (*n* = 78) having longitudinal tau‐PET data. As an independent sample, we further included regional post‐mortem tau stainings from 93 atypical AD patients from two sites (*n* = 19 PCA‐AD, *n* = 32 lvPPA‐AD, *n* = 23 bvAD, *n* = 19 CBS‐AD). Gaussian mixture modeling was used to harmonize different tau‐PET tracers by transforming tau‐PET standardized uptake value ratios to tau positivity probabilities (a uniform scale ranging from 0% to 100%). Using linear regression, we assessed whether 1) brain regions with stronger functional connectivity showed greater covariance in cross‐sectional and longitudinal tau‐PET and post‐mortem tau pathology, and 2) functional connectivity of tau‐PET epicenters and tau‐PET accumulation epicenters was associated with cross‐sectional and longitudinal tau patterns.

**Result:**

Tau‐PET epicenters—defined as the 5% brain regions with the highest tau load—aligned with clinical variants, e.g. a posterior pattern in PCA‐AD (“visual AD”) and left‐hemispheric temporal predominance in lvPPA‐AD (“language AD”) (Figure 1). More strongly functionally connected regions showed correlated concurrent tau‐PET levels, which was confirmed with post‐mortem data (Figure 2). Moreover, the connectivity profile of tau‐PET epicenters and accumulation epicenters corresponded to tau‐PET progression patterns (Figure 3).

**Conclusion:**

Our data are consistent with the hypothesis that tau propagation occurs along functional connections originating from local epicenters, across all AD clinical variants. Since tau proteinopathy is a key driver of neurodegeneration and cognitive decline, this finding may advance personalized medicine and participant‐specific endpoints in clinical trials.